# The skin microbiome of *Xenopus laevis* and the effects of husbandry conditions

**DOI:** 10.1186/s42523-021-00080-w

**Published:** 2021-02-05

**Authors:** Maya Z. Piccinni, Joy E. M. Watts, Marie Fourny, Matt Guille, Samuel C. Robson

**Affiliations:** 1 School of Biological Sciences, University of Portsmouth, Portsmouth, UK; 2 European Xenopus Resource Centre, University of Portsmouth, Portsmouth, UK; 3 Centre for Enzyme Innovation, University of Portsmouth, Portsmouth, UK; 4 University of Rouen-Normandy, Rouen, France; 5 School of Pharmacy and Biomedical Sciences, University of Portsmouth, Portsmouth, UK

**Keywords:** Cutaneous microbiome, *Xenopus laevis*, Husbandry, Frog skin microbiota, Tadpoles, Illumina MiSeq, 16S rRNA amplicon

## Abstract

**Background:**

Historically the main source of laboratory *Xenopus laevis* was the environment. The increase in genetically altered animals and evolving governmental constraints around using wild-caught animals for research has led to the establishment of resource centres that supply animals and reagents worldwide, such as the European *Xenopus* Resource Centre. In the last decade, centres were encouraged to keep animals in a “low microbial load” or “clean” state, where embryos are surface sterilized before entering the housing system; instead of the conventional, “standard” conditions where frogs and embryos are kept without prior surface treatment. Despite *Xenopus laevis* having been kept in captivity for almost a century, surprisingly little is known about the frogs as a holobiont and how changing the microbiome may affect resistance to disease. This study examines how the different treatment conditions, “clean” and “standard” husbandry in recirculating housing, affects the skin microbiome of tadpoles and female adults. This is particularly important when considering the potential for poor welfare caused by a change in husbandry method as animals move from resource centres to smaller research colonies.

**Results:**

We found strong evidence for developmental control of the surface microbiome on *Xenopus laevis*; adults had extremely similar microbial communities independent of their housing, while both tadpole and environmental microbiome communities were less resilient and showed greater diversity.

**Conclusions:**

Our findings suggest that the adult *Xenopus laevis* microbiome is controlled and selected by the host. This indicates that the surface microbiome of adult *Xenopus laevis* is stable and defined independently of the environment in which it is housed, suggesting that the use of clean husbandry conditions poses little risk to the skin microbiome when transferring adult frogs to research laboratories. This will have important implications for frog health applicable to *Xenopus laevis* research centres throughout the world.

**Supplementary Information:**

The online version contains supplementary material available at 10.1186/s42523-021-00080-w.

## Background

The South African clawed frog (*Xenopus laevis*), is a model species utilised worldwide, first used in the development of pregnancy tests [1, 2]. Their availability, together with their ability to generate many large embryos throughout the year, marked their establishment as a research model for developmental biology, biochemistry and genetics [3–5]. It is now well understood that animal systems exist as holobionts [6], with a complex associated microbial community affecting host health and function [7–9]. Amphibian skin microbiota are highly diverse, with Western chorus frogs (*Pseudacris triseriata*) and Northern leopard frogs (*Lithobates pipiens*) showing similar levels of diversity to those detected on human skin [10].

Despite the global use of this model system, little attention has been given to understanding how the microbiome affects frog health and how they respond to stress [11, 12]. Skin-related microbial communities influence host resistance to infection, as reviewed in Ross et al (2019) [13]. In amphibians, skin plays an important role in respiration, thermoregulation, osmoregulation, pigmentation and protection from predators and pathogens [14–17]. It is characterized by a thin mucosal surface produced by granular glands that secrete oils and other substances [14, 15], and due to its moist nature sustains a wide microbial community composed of bacteria, archaea, fungi and protozoans [18, 19]. Studies on the skin microbiota of amphibians have highlighted the link between microbiome species richness with their ability to resist pathogens and overall health [20–25].

The increased popularity of *Xenopus* in the last decade has led to development of resource centres providing not only animals but also support, training and *Xenopus*-specific resources [26]. The two main centres, the National *Xenopus* Resource (NXR, USA) and the European *Xenopus* Resource Centre (EXRC, UK), each hold more than 10,000 animals including hundreds of genetically altered (GA) lines. Historically, *Xenopus* were kept in tanks holding up to 100 animals with water changes two or three times each week [12]. More recently, many laboratories have adopted recirculating systems, in which water is changed gradually and treated before re-entering the system [12]. This increase in recirculating systems, together with the risk of rare but devastating infections causing the loss of entire *Xenopus* colonies [27] and increased animal movement between research establishments [28], has increased the need for improved biosecurity and a move towards “clean” surface-sterilized colonies [29]. This process is achieved by exposure of early embryos to thimerosal and then to ethanol, and whilst called sterilization, it is possible that some microorganisms persist after the treatment [29]. However, such housing conditions divert considerably from the frogs’ natural habitats, which include swamps, dams and forest pools [30], suggesting a need to understand the consequences of such housing on the frogs’ health and their ability to resist infection.

Due to an increase in amphibian infectious diseases, specifically *Batrachochytrium dendrobatidis* (Bd), several amphibian microbiomes have been described using culture-dependent methods [21, 31–34], and next-generation sequencing (NGS) [10]. Due to the issues with non-culturability [35, 36], further studies have focused on NGS to characterise skin microbial communities of different amphibian species [14, 20, 22, 37–42], with particular emphasis on the antimicrobial properties of the amphibian skin mucosa and how this is vital for the control of infectious diseases [7, 23, 39]. Comparisons between different species and diverse habitats indicate that both the quantity and diversity of bacteria are higher when isolated from wild populations of amphibians than when isolated from those kept in captivity [40, 43, 44].

In this study, we investigate how the skin microbiome changes between the two most commonly used *Xenopus laevis* husbandry conditions, “standard” and “clean” housing, using 16S rRNA amplicon sequencing. Moreover, we analysed the microbiome of adult skin (18-month-old females), tadpole skin (~ 1-month-old), and their housing water to characterise the animal’s skin microbiota and environment at different developmental stages. A better understanding of the *Xenopus laevis* microbiome and its interactions with its environment has important implications for frog husbandry in resource centres and the laboratories depending on them. It may also inform future conservation efforts for endangered amphibia.

## Results

**Fig. 1:**
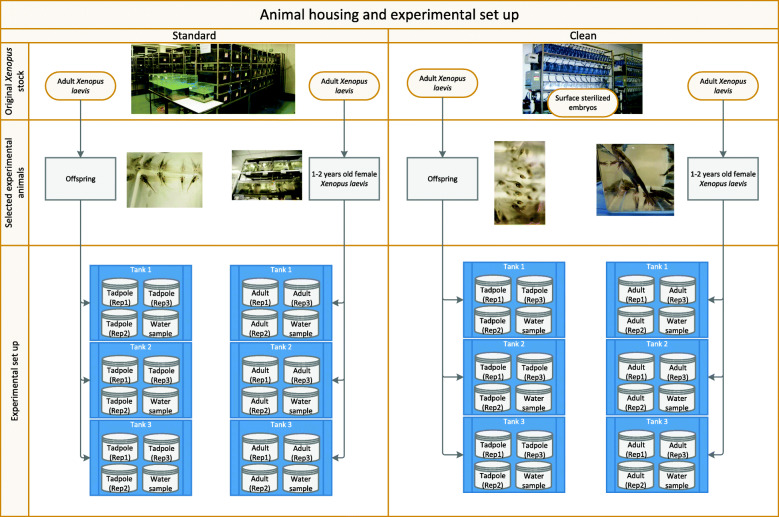
Experimental design. Schematic outlining the housing conditions, developmental stages, and experimental set up used in this experiment

**Fig. 2:**
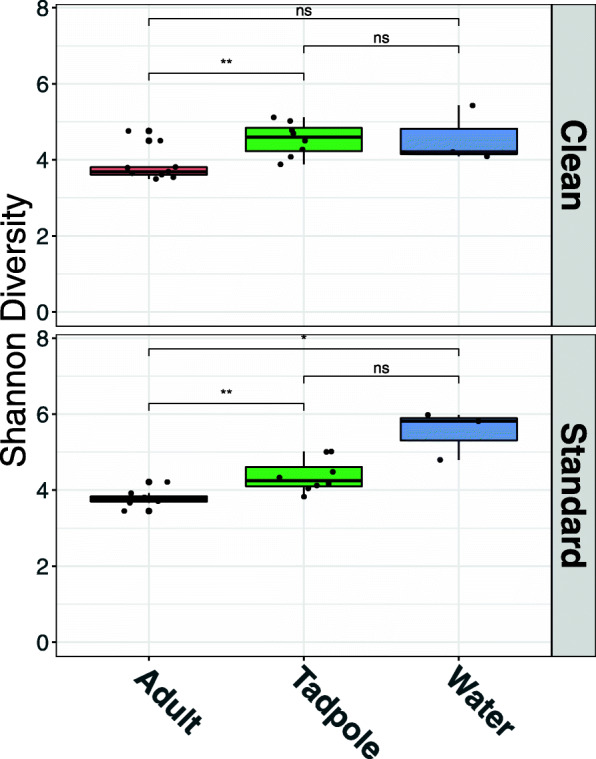
Alpha diversity across the data set. Alpha diversity (represented by the Shannon Index) across the data set, representing the diversity of species within each sample group. For each sample, the alpha diversity was calculated based on 10 random subsamples of 5000 OTUs. Significant differences between groups are based on an independent two-sample t-test (** = *p* ≤ 0.01; * = *p* ≤ 0.05; ns = not significant). Full alpha diversity values for each individual sample, including observed diversity and Chao1 Index, can be found in Supplementary Table 1

The experimental set up outlining the housing conditions and developmental stages used throughout are shown in Fig. 1. Alpha diversity was compared between samples (Fig. 2; Supplementary Table 1) and highlighted differences in bacterial community composition between conditions. Alpha diversity of species was estimated using both the observed diversity and the Shannon Index. Overall, Shannon bacterial diversity appears to be higher for tadpoles than for adult frogs for both standard (t = − 3.39; df = 10.3; *p* = 0.007) and clean (t = − 3.11; df = 14.8; p = 0.007) conditions. However, no significant difference in overall Shannon diversity was observed between clean and standard conditions for adult frogs (t = 0.51; df = 11.9; *p* = 0.616) or tadpoles (t = 0.60; df = 14.0; *p* = 0.560).

**Fig. 3:**
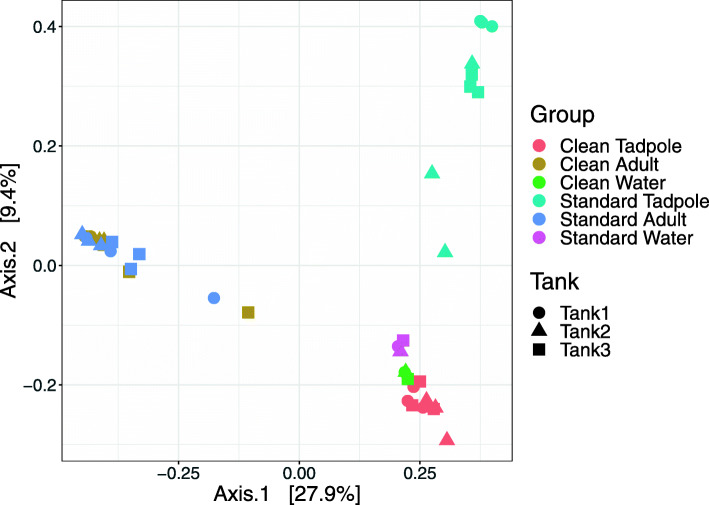
Principal coordinates analysis (PCoA) of all samples in the data set. Axis 1 and Axis 2 represent the coordinates of the greatest sources of orthogonal variation within the data and represent 27.9 and 9.4% of variation in the entire data set respectively. Whilst results from biological replicates and distinct tanks are generally consistent, Axis 1 represents the difference in diversity between the adult frogs as compared to both tadpoles and environmental water samples. Axis 2 shows close clustering between adults housed in standard and clean conditions, and tadpoles and their environmental water samples for clean conditions only. However, a significant change in diversity is seen for tadpoles from standard conditions, despite water samples from standard tanks showing similar profiles to those from clean tanks

Principal coordinates analysis (PCoA) was used to explore the beta diversity to better understand the overall variation in diversity between conditions and within tank variability (Fig. 3). The PCoA analysis identified a clear difference between the bacterial diversity present on the skin of adult frogs as compared to that present on tadpoles and in the water in which the animals live. From Fig. 3 it can be observed, that adults in standard and clean conditions cluster very closely together, indicating that they have very similar bacterial species composition. However, tadpoles appear to have a more distinct profile, seemingly more dependent on their environmental conditions. Tadpoles within the clean conditions show very close clustering to the water samples from the corresponding tanks. Conversely, tadpoles from standard conditions seem to have a much more distinct bacterial microbiome composition when compared to their associated tank water, with high individual variability detected. This was confirmed via a 2-way permutational multivariate analysis of variance (PERMANOVA) analysis which identified a highly significant effect on diversity between adult, tadpole and water samples (F = 10.71; df = 2; *p* = 0.0001), a significant effect due to the housing conditions (F = 2.84; df = 1; *p* = 0.0056), but also a significant interaction effect due to the fact that diversity in tadpoles differs significantly between clean and standard conditions whilst it remains consistent in adults (F = 2.69; df = 2; *p* = 0.0011).

A number of unique OTUs and corresponding taxonomic annotation were identified within each of the sample groups, with tadpoles showing roughly 3-times higher numbers of unique OTUs than their adult counterparts (Table 1). Interestingly, whilst water from standard tanks contained more than twice the number of unique OTUs than water from clean tanks, adult frogs showed very similar numbers of unique OTUs in both conditions, while tadpole analysis detected more unique OTUs from samples in the clean tanks compared to standard treatment.

**Table 1:** Unique taxa detected at multiple different taxonomical levels

Group	Phyla	Classes	Orders	Families	Genera	Species	OTUs
Clean tadpole	15	25	39	54	60	42	921
Clean adult	12	19	23	35	27	20	295
Clean water	14	26	40	54	47	28	446
Standard tadpole	12	22	32	44	43	35	705
Standard adult	10	20	25	35	25	19	292
Standard water	19	38	60	82	94	68	1146

This table shows the total number of unique taxa identified at least once for each condition at each specific taxonomic level

**Fig. 4:**
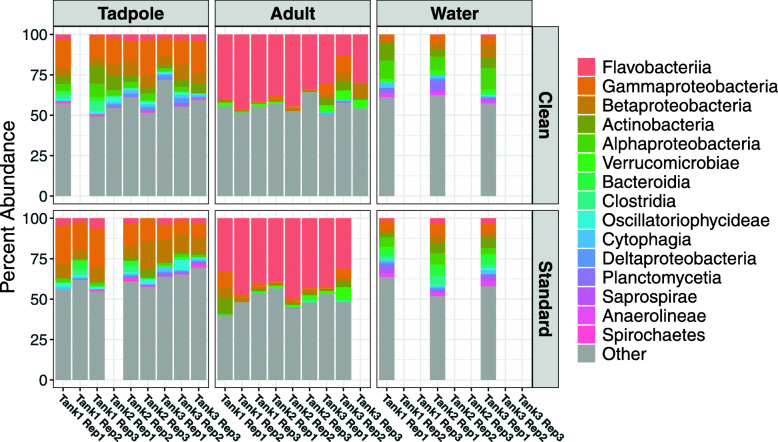
Barplot showing the distribution of bacteria at the phylum level of taxonomy. In each case, the top 15 phyla are highlighted, with all remaining phyla shown in grey

Analysis of the distribution of the numerically dominant 15 taxonomic classes of bacteria detected on tadpoles (Fig. 4; left-hand panels) shows a more varied microbiome than that of the adults, consisting of relatively high detection of Gammaproteobacteria (15.5 and 14.6% in clean and standard samples respectively) and Betaproteobacteria (7.1 and 10.6% for clean and standard samples respectively), along with other bacterial classes also found within their water environment. In contrast, the adult frog microbiome (Fig. 4; middle panels) is dominated by a single class of bacteria; Flavobacteria (35.2 and 40.1% for clean and standard samples respectively). No other bacterial class accounts for more than 3% of the remaining diversity. This dominance seems to be independent of the housing conditions, as Flavobacteria are detected only in low levels, in the water samples (Fig. 4; right-hand panels) from both standard housing (2.6%) and clean conditions (0.6%).

**Fig. 5:**
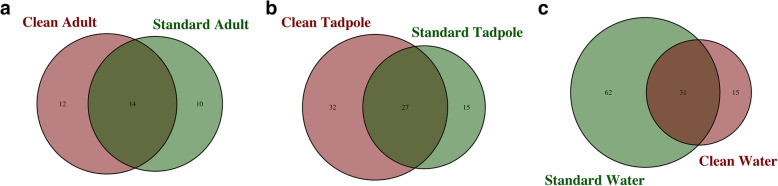
Comparison between genera identified on adult frogs, tadpoles and in water controls. Venn diagrams showing the overlap between unique genera identified in samples from standard conditions compared to clean conditions for **a**) adult frogs, **b**) tadpoles and **c**) water samples

The most common genus detected on adult skin tissue had high sequence similarity to Bergeyella, accounting for 18.8 and 19.6% of sequence abundance in frogs from the clean and standard samples respectively (Supplementary Figure 3). In contrast, this genus is very low in tadpoles (< 0.2%) and almost entirely undetectable in environmental water samples from both clean and standard tanks. In tadpoles, sequences most closely related to Alkanindiges are the numerically dominant genus in standard samples, accounting for 8.6% of all genera. This is most notable in Tank 1, suggesting a possible tank-specific bias. However, Alkanindiges-like sequences appear to be largely absent on clean-housed tadpoles (< 0.5%). Instead, a larger proportion of sequences with high similarity to Pseudomonas are present, along with very small proportions of a large number of other genera. The overlap between the unique genera identified in samples from both clean and standard conditions for adult frogs, tadpoles and water samples can be seen in Fig. 5. Interestingly, whilst more unique genera are identified in standard water (Fig. 5c), it is the microbiome of tadpoles housed under clean conditions that appears to show the greatest diversity (Fig. 5b).

**Fig. 6:**
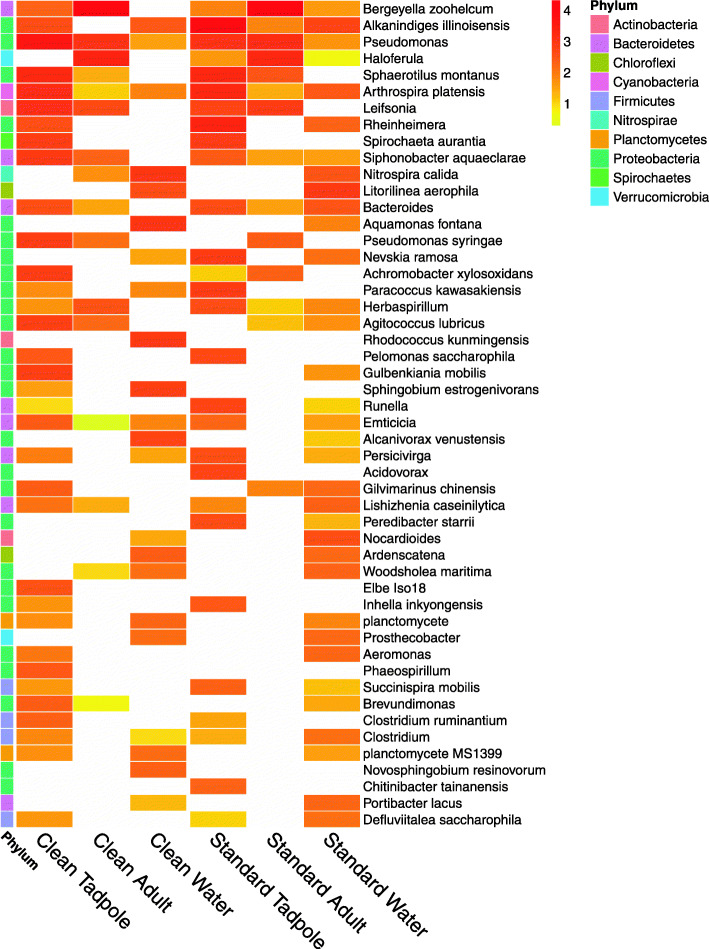
Heatmap showing the top 50 species identified across the data set. OTU counts from biological replicates amongst the different replicate tanks were combined to give a single count, which was normalised so that all samples had the same total. Cells are coloured based on the percentage of the total OTU count (on a log10 scale), with more abundant OTUs shown in red

**Fig. 7:**
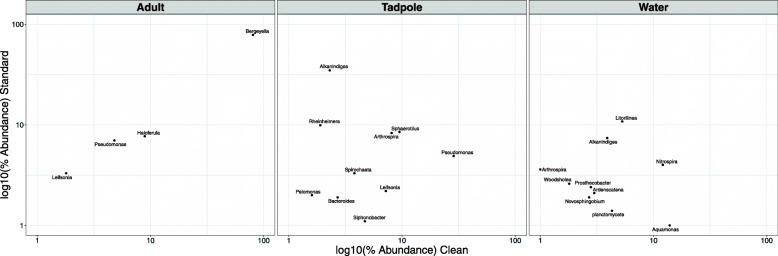
Relative abundance (%) of core microbiome members shared between clean and standard housing conditions for adult, ii) tadpole, and iii) environmental water samples. OTUs were combined at the Genus level and were identified as core microbiome members based on an average relative abundance greater than 1% in both Clean and Standard conditions. Comparative relative abundance (on a log10 scale) is plotted for clean and standard conditions to highlight similarities and differences between the housing conditions

The 50 most abundant species identified by their similarity to sequences detected across the data set are shown in Fig. 6. The most common sequence detected has high similarity to *Bergeyella zoohelcum* and is almost exclusively identified on adults, with nearly 500-times lower abundance identified in the corresponding water sample for standard samples, and zero abundance identified in clean water samples. This suggests that the species, represented by these OTUs are able to colonise the adult frog skin, independent of environmental conditions. In comparison, *Bergeyella zoohelcum* is seen only at low levels on tadpoles. Instead, sequences similar to *Alkanindiges illinoisensis* are identified at high levels on tadpoles in standard conditions, but show 20-fold decreased abundance in clean samples. Conversely, sequences with high similarity to *Pseudomonas* are identified on clean tadpoles, but are almost 4-times less abundant on tadpoles from standard conditions. Figure 7 shows the relative abundance of the core microbiome members shared by both clean and standard housing conditions for adults and tadpoles. OTUs were combined at the Genus level to match results shown in Supplementary Figure 3. Almost all are annotated as *Bergeyella* for adults, with *Haloferula*, *Pseudomonas,* and *Leifsonia* also present albeit at lower levels. Interestingly, in adults the proportion of each of these four microbiome members remains constant between clean and standard conditions. In contrast, the tadpole microbiome is quite distinct from the adult, and while clean tadpoles were dominated by *Pseudomonas*, the standard tadpoles were dominated by *Alkanindiges*. The phylogeny of OTUs in the 20 distinct genera identified as being abundant above 1% in both clean and standard conditions for any sample group is shown in Supplementary Figure 4.

Whilst the core adult microbiome is clearly extremely similar whether produced in a clean or standard environment, the same is not the case for tadpoles. The 15.1-fold difference in *Alkanidiges* abundance and 5.8-fold difference in *Pseudomonas* between the environments in tadpole samples are extremely striking but do not appear simply to reflect the abundance in their environments. In the water samples, *Alkanindiges* OTUs are only 1.9-fold higher in standard conditions, whilst *Pseudomonas* show similar, low levels in both clean (0.5%) and standard (0.6%) water samples. In contrast, *Arthospira* OTUs are 3.6-fold higher in standard water than clean, but show similar levels of 8% on tadpole skin. Generally, these results highlight clear differences between skin microbiota for adult frogs and tadpoles, with few significant differences between the clean and standard conditions beyond those of the most abundant species. To identify whether bacteria associated with adult skin microbiome have inhibitory effects on the pathogen *Batrachochytrium dendrobatidis*, of which *Xenopus laevis* is a notorious carrier, sequences detected in this current study were compared to 1127 bacterial species with putative inhibitory regulation identified in a previous study performed by Woodhams et al (2016) [44]. OTU sequences from the present study were compared with these 1127 bacterial genome sequences using BLAST [45] to identify bacteria present in the dataset that may represent the same species, based on 99% sequence overlap with an e-value less than 1e-100. This comparative analysis identified 43 unique OTUs meeting these criteria, primarily clustering amongst 5 genera. The abundance was low for all of the individual OTUs (maximum of 0.7%), so these specific OTUs are not amongst the most abundant OTUs detected in the dataset. Whilst Alkanindiges and Pseudomonas are amongst the most abundant tadpole-associated genera, particularly in the standard samples, the Bd-inhibitors identified are not amongst those most highly abundant in the tadpole skin microbiome. While we do see increased abundance for one Pseudomonas-like OTU in the standard tadpoles and standard water (compared to clean), this is not a general trend suggestive of enrichment for genera with Bd-inhibitory properties.

## Discussion

There have been several studies focussing on amphibians examining culture-dependent microbiome analysis [21, 31–34, 46]. However, advances in sequencing technologies have allowed investigation of the links between the host and the microbial flora that live within the animal (as either symbiont, commensal or parasite) using culture-independent methods (e.g. 16S rRNA amplicon sequencing) [38, 41, 43, 47]. The culture-independent studies reported here provide vital information for a more detailed understanding of the *Xenopus laevis* skin microbiome.

This present study indicates that tadpoles of the same genetic background, but kept in different housing conditions, have distinct skin microbiomes influenced in some way by their environment. However, the analysis of adults indicates highly similar microbial communities, indicative of convergent skin microbiome development. The adult skin microbiomes are largely independent of their housing conditions and include some bacterial species that are undetectable in samples of the tank water in which they live. These data from laboratory reared, adult *Xenopus laevis* agree with the findings of studies on different species of amphibians in a range of environments; they support the hypothesis that many adult amphibians can selectively enrich their microbiome with species of bacteria that are rare in their environment [7, 16, 41, 44, 48, 49].

Our results support the skin surface of *Xenopus laevis* being a highly selective environment and that bacteria with suitable adaptations to these conditions can form a strong, possibly symbiotic association. From this study it appears that there is a substantive difference in the skin during development and a stable microbiome appears late in the larval stage or at metamorphosis. Using amphibians from the environment McKenzie et al (2012) [10]observed that in three different amphibian larvae from the same geographical site the skin microbiome did not resemble that of the environmental microbial community and differed between species. However, major differences exist between this environmental study and this current laboratory-based analysis – it is a possibility that the ability to control the microbiome develops during larval growth and may vary between species, something that further laboratory study in *Xenopus* and other amphibian species could determine. Nonetheless, the developmental changes that we observe between the microbiome of tadpoles and adults are supported by data obtained in the wild [18, 21, 48, 50]. Previous studies have postulated that much of the reason for this developmental change of the microbiome is the move from an aquatic to primarily terrestrial life that accompanies metamorphosis [48]. However, this is not supported by our study as *Xenopus laevis* remain aquatic as adults. Our data are also inconsistent with the theory proposed by Ellison et al (2018) [21]: that the skin of metamorphosing frogs is initially colonized by a relatively simple microbial community, but this community grows in complexity as the frog reaches adulthood. However, our results determined that the adult microbiome is dominated by a small number of bacterial species. Major changes occur to the skin during frog metamorphosis: the tadpole epidermis consists of two or three layers of cells contacting a collagen lamella and the dermis of that lamella, a single layer of fibroblasts and melanocytes. At metamorphic climax the outer two epidermal cell layers die and the subepithelial fibroblasts invade the collagen lamella, the dermis becomes thicker and the adult skin glands form [51]. This complete change in the structure and physiology of the skin is most likely to underlie the concomitant major change in the microbiome. More difficult is understanding the mechanisms generating the microbiome change. How amphibia regulate their skin microbiome is still largely unknown, however in Eastern Hellbenders there is evidence that microbiome richness correlates positively with the population genetic diversity of the host, implying host-dependent control of the microbiome [52]. This observation is however not supported by an elegant study of whether the genetic distance between different clades of salamander species in California correlate with their microbiome, or if their environment is a better predictor. In this work environment dominated over the relatedness of the animals [53]. Although these two studies take different approaches, the lack of a simple explanation for what controls the amphibian microbiome is clear.

The adult microbiome relative abundance analysis was dominated by sequences with the most 16S rRNA sequence similarity to representatives in the *Bergeyella* genera, (18.8% in clean conditions and 19.6% in standard conditions). *Bergeyella, Chryseobacterium* and *Riemerella* form a separate branch within the *Bacteriodetes* phylum and have a complicated phylogeny [54] that has recently been comprehensively revised. A number of reports detail novel species detected from these genera on amphibian and fish skin [16, 54–56] and they are often reported as opportunistic pathogens. However, a recent culture-based study on *Xenopus laevis* found [46] two ultrasmall *Chryseobacterium* strains and due to their strong attachment to the skin suggested these are epibonts with complex attachment structures to assist tight binding to the skin surface. Interestingly, sequences with similarity to Bergeyella were not detected in the tadpole analysis, instead OTUs with high similarity to Pseudomonas and Alkanindiges species were the most abundant detected. They are both gram-negative Gammaproteobacteria and commonly found in amphibian and frog microbiome community profiling studies [8, 14, 57, 58].

The frogs used in this investigation came from a colony in which Bd is very seldom discovered at detectable levels. Nonetheless our previous data show that it is not absent and that some of the animals may carry it, albeit undetectably [59], so there may be an element of selection for Bd-inhibitor organisms in the skin microbiome. From our analysis of potential Bd-inhibitors within the microbiome of adult frogs and tadpoles there was a slight enrichment for genera with Bd-inhibitory properties, however as these relative abundances are not quantitative any conclusions would require further studies to be performed. Nonetheless, there is clear potential for laboratory studies to inform conservation biology in the wild.

A range of studies have linked amphibian health and the ability of animals to resist disease to their skin microbiome. For example, unhealthy amphibians often show low microbial diversity, species richness, species phylogenetic diversity and species evenness compared to healthy animals [21]. The use of Probiotic treatments in captive amphibian has been reported to increase the resistance/tolerance of these species to infections [44]. Moreover, the use of a multi-species probiotic was reported to have increased efficiency against infection [33] and some studies confirm that after treatment they found no frogs with high pathogen levels present and high numbers of inhibitory bacteria [20]. There are clearly many possible microbiome-based interventions with the potential to improve welfare and experimental reproducibility in this important model organism.

There may be important lessons to be learned from studying the tadpole microbiome for *Xenopus* welfare and for experiments using this model, particularly in immunology. In the Cuban tree frog (*Osteopilus septentrionalis*), reduced exposure to environmental microbes as tadpoles had a profound effect on the long-term immune system of the frog, but the microbiota of adults frogs was not affected by pathogen exposure [37]. This finding was supported by Walke et al (2014) [41], who also took into account the theoretical vertical gene transfer and its effects on the symbiont. In laboratory *Xenopus,* embryos are normally kept with a supplement of antibiotics for the first several days of their life, independent of their housing condition. The effect of this needs to be investigated further, since we do not currently know the long-term effects of such treatment; it is possible that this practice reduces the immunocompetence of laboratory *Xenopus* in a similar way to the Cuban tree frog. Additionally, it is clear that for other laboratory animals [60, 61], the gut microbiota can influence experimental results [62, 63]. Microbiome-dependent irreproducibility seems a low risk for the studies of embryos that *Xenopus* are most often used for. However, the use of *Xenopus laevis* for analysing rare human genetic diseases is increasing rapidly, meaning that researchers are now investigating events later in *Xenopus* development. In such studies, the risks of such potential variation may rise.

## Conclusion

This study suggests that the microbiome of adult *Xenopus* is subject to rigorous environmental selection resulting in a stable and defined microbial community independent of laboratory husbandry conditions. The highly similar microbiomes of adult female *Xenopus* from clean and conventional housing strongly suggests that using clean husbandry in the resource centres provides little or no additional risk to frog health when they are moved to research laboratories. However, the tadpole microbiome is more variable and subject to changes in environmental conditions. Further studies should be performed to better understand the complex relationship between the skin microbiome composition and the long-term outcomes for tadpole and frog health reared in the laboratory.

## Methods

### Housing conditions

*Xenopus laevis* frogs were kept in recirculating water in Tecniplast/Marine Biotech systems in which the water was treated with a UV lamp and particulate, mechanical and carbon filters. The water temperature was kept at 18.5 °C (range 18–19 °C), the conductivity at 1550 μs/cm (range 1500–1600 μs/cm, maintained by Tropic Marin/Instant Ocean sea salt dosing), the pH at 7.5 (range 7–8, maintained using sodium bicarbonate dosing). The input water was charcoal filtered Portsmouth mains water. Frogs were housed in 23 l tanks at a stocking density of 7 adult females per tank. The flow rate of the recirculating water was 1.33 l per minute in adult tanks and minimal flow in tadpole tanks. The light cycles were 13 h of fluorescent light and 11 h dark with night light. Tanks were supplemented with environmental enrichment of black plastic guttering and downpipe tubes cut in half in 20 cm lengths. Adult frogs were fed Horizon 23 trout pellets 2–3 times per day, a few pellets each. Tadpoles were fed Sera-micron algae daily with water flow turned to a slow drip. All animals were fed 5 days per week.

Six tanks within a 9-tank rack were designated randomly for the experiment in a “clean” room and a “standard” room. The remaining tanks were undisturbed. Three tanks per clean and standard rack were populated with adult female *Xenopus laevis*, and three tanks with 4-week old tadpoles (Fig. 1). The adults used originated from the same clutch of eggs, as did the tadpoles and so each group consisted of siblings of the same age. In common with most laboratory-bred *Xenopus*, both the tadpoles and adults spent the first week of their lives in buffer supplemented with penicillin and streptomycin. All animals used in this study appeared to be in excellent health.

### Sample collection

Adult *Xenopus laevis*, pre-metamorphic tadpoles and tank water were analysed. We focused on females since many laboratories do not keep male frogs due to the successful introduction of frozen sperm and this decision was supported by the fact that no statistical difference between male and female skin microbiomes was found in the Red-eye tree frog [40]. Water (50 mL) was collected in sterile Falcon tubes from each of the designated tanks. Adult *Xenopus laevis* were taken from their tanks, rinsed by submersion in sterile distilled water to remove transient bacteria and swabbed with a sterile cotton swab (Medical Wire Equipment Co, MW-100) before being placed back in the system. To swab thoroughly and consistently, the same swabbing technique was followed for each sample and new nitrile gloves were used between samples. The pattern consisted of ten strokes on the ventral side, ten strokes on the dorsal side, five strokes under each front leg and five strokes between the toes. Tadpoles were trapped with a net, rinsed in distilled water and swabbed ten times on the dorsal side and ten times on the ventral side. Swabs were kept at 4 °C for up to 1 week before DNA preparation.

### Sample preparation

Water samples were filtered through a 0.45 μM Cellulose Nitrate Filter (Sartorius). These filters and the cotton swabs were suspended in 500 μL of phosphate buffer saline (PBS) for 5 min with gentle agitation, 200 μL of this solution was removed and mixed with 20 μL of proteinase K (20 mg/ml). An outline of the sample preparation process can be seen in Supplementary Figure 1.

### DNA extraction and 16S rRNA gene sequencing

Each swab was processed following the DNeasy Blood and Tissue Kit (QIAGEN) manufacturer’s instructions. The extracted DNA was quantified by spectrophotometry and shipped to LGC Genomics GmbH. The 16S rRNA gene was targeted by PCR with primers 341F/785R used by Klindworth et al, 2013 [64] (341F 5′-CCTACGGGNGGCWGCAG-3′ and 785R 5′-GACTACHVGGGTATCTAATCC-3), samples pooled, library prepared (incl. Indexing*2) and quality controlled by gel electrophoresis before cluster generation and sequencing using Illumina MiSeq V3.

### Sequence filtering and OTU calling

The quality of the raw read data was assessed using FastQC [65] v0.11.6 to ensure no consistent sequencing artefacts were present in the data. Contamination was assessed by mapping against a panel of 32 genomes, including large and small subunit rRNAs, bacterial and viral genomes, and genomes from a wide range of species (including human) using fastq-screen v0.13.0 (https://www.bioinformatics.babraham.ac.uk/projects/fastq_screen/). Reads were trimmed to remove adapter sequences and poor quality reads using trim_galore v0.5.0 [66] with parameters “--illumina -q 20 --stringency 5 -e 0.1 --length 20 --trim-n” (https://www.bioinformatics.babraham.ac.uk/projects/trim_galore/). Combination of paired-end reads, denoising and identification of operational taxonomic units (OTUs) was performed using Qiime2 version 2019.4.0 [67]. Taxonomy of identified OTUs was assigned based on the SILVA v132 database [68] with 99% similarity. The number of raw read pairs, filtered read pairs, and OTUs identified for each sample can be found in Supplementary Table 2. Full annotation and total counts for each identified OTU across the individual samples can be found in Supplementary Table 3.

Further analyses of the Qiime2 output data was conducted in R, primarily using the phyloseq package [69]. Samples with fewer than 5000 OTUs were filtered out from further analysis, as were OTUs not present in any sample. Read depth above 5000 OTUs was considered sufficient to capture the vast majority of diversity based on asymptotic convergence of the rarefaction curve (Supplementary Figure 2). OTU counts for each sample were normalized to the library size to give proportional abundance as percentage values. Samples within groups were combined by adding the OTU counts across all samples and normalizing to the total library size to give percentage values within groups. Alpha diversity was assessed using both Shannon index [70] and observed diversity (the number of distinct OTUs identified across each data set). Comparison of diversity between groups was assessed based on an independent two-sample t-test. Principal coordinates analysis (PCoA) plots were produced based on the Bray-Curtis dissimilarity measure [71] using the phyloseq package [69] in R to observe beta diversity. OTUs showing significant differences in levels between conditions were identified using the DESeq2 package in R [72], by scaling the normalized abundance proportions to the median depth of coverage overall samples to produce integer counts comparable between samples. *P* values were adjusted for multiple testing using the Benjamini and Hochberg false discovery rate correction [73]. Significantly changing OTUs were identified as those with a fold change greater than 2 (up or down) and an adjusted *p*-value less than 0.05. Representative figures were generated using the ggplot2 [74] and pheatmap [75] packages in R. The effect of housing conditions (standard or clean) and sample source (adult, tadpole or frog) on bacterial diversity was assessed using permutational multivariate analysis of variance (PERMANOVA) analysis with the adonis2 function from the vegan [76] package in R, using Bray-Curtis dissimilarity and 9,999 permutations.

## Supplementary Information

**Additional file 1: Supplementary Figures:** Additional figures.

**Additional file 2: Supplementary Table 1**: Alpha diversity across the data set. Three measures of the alpha-diversity (observed diversity, Chao1 Index and Shannon Index) across the data set, representing the diversity of species within each sample. For each sample, random subsamples of 5000 OTUs were taken for each sample and alpha-diversity scores were calculated. Alpha diversity scores are shown by mean +/− standard deviation over 10 random subsamples.

**Additional file 3: Supplementary Table 2**: Statistics for 16s rRNA amplicon sequencing. The number of raw read pairs, adapter-trimmed read pairs, and unique OTUs identified by QIIME2 for each sample are shown.

**Additional file 4: Supplementary Table 3**: OTU-level annotation and counts. For each unique OTU identified by QIIME2, complete annotation assigned from SILVA and counts for each individual sample are shown.

**Additional file 5: Supplementary Table 4**: Core microbiome shared between clean and standard conditions. Relative abundance (%) of core microbiome members shared between clean and standard housing conditions for adult, ii) tadpole, and iii) environmental water samples. OTUs were combined at the Genus level and were identified as core microbiome members based on an average relative abundance greater than 1% in both Clean and Standard conditions. Log2FC represents the log2 fold change of abundance in the clean samples compared to that in the standard samples.

## Data Availability

Raw sequencing data were deposited in the NCBI Sequence Read Archive (SRA) under BioProject accession code PRJNA622548 (https://www.ncbi.nlm.nih.gov/bioproject).

## Abbreviations

BBSRC: Biotechnology and Biological Sciences Research Council
Bd: *Batrachochytrium dendrobatidis*
DNA: Deoxyribonucleic acid
E3: Expanding Excellence in England
EXRC: European *Xenopus* Resource Centre
GA: Genetically altered
NGS: Next-generation sequencing
NXR: National *Xenopus* Resource
OTU: Operational taxonomic units
PBS: Phosphate buffer saline
PCoA: Principal coordinates analysis
PERMANOVA: Permutational multivariate analysis of variance
RNA: Ribonucleic acid
rRNA: Ribosomal RNA
SPF: Specific pathogen-free
SRA: Sequence Read Archive

## References

[1] Gurdon JB, Hopwood N (2000). The introduction of Xenopus laevis into developmental biology: of empire, pregnancy testing and ribosomal genes. Int J Dev Biol.

[2] Elkan ER. The Xenopus pregnancy test. Br Med J. 1938;2:1253–6. 10.1136/bmj.2.4067.1253 PMC2211252 20781969

[3] Tandon P, Conlon F, Furlow JD, Horb ME (2017). Expanding the genetic toolkit in Xenopus: approaches and opportunities for human disease modeling. Dev Biol.

[4] Harland RM, Grainger RM (2011). Xenopus research: metamorphosed by genetics and genomics. Trends Genet.

[5] Segerdell E, Bowes JB, Pollet N, Vize PD (2008). An ontology for Xenopus anatomy and development. BMC Dev Biol.

[6] McFall-Ngai M, Hadfield MG, Bosch TCG, Carey HV, Domazet-Lošo T, Douglas AE, Dubilier N, Eberl G, Fukami T, Gilbert SF, Hentschel U, King N, Kjelleberg S, Knoll AH, Kremer N, Mazmanian SK, Metcalf JL, Nealson K, Pierce NE (2013). Animals in a bacterial world, a new imperative for the life sciences. Proc Natl Acad Sci U S A.

[7] Loudon AH, Woodhams DC, Parfrey LW, Archer H, Knight R, McKenzie V, Harris RN (2014). Microbial community dynamics and effect of environmental microbial reservoirs on red-backed salamanders (plethodon cinereus). ISME J.

[8] Woodhams DC, Vredenburg VT, Simon MA, Billheimer D, Shakhtour B, Shyr Y, Briggs CJ, Rollins-Smith LA, Harris RN (2007). Symbiotic bacteria contribute to innate immune defenses of the threatened mountain yellow-legged frog, *Rana muscosa*. Biol Conserv.

[9] Jiménez RR, Sommer S (2017). The amphibian microbiome: natural range of variation, pathogenic dysbiosis, and role in conservation. Biodivers Conserv.

[10] McKenzie VJ, Bowers RM, Fierer N, Knight R, Lauber CL (2012). Co-habiting amphibian species harbor unique skin bacterial communities in wild populations. ISME J.

[11] Tinsley R (2010). Amphibians, with special reference to xenopus. The UFAW handbook on the care and management of laboratory and other research animals.

[12] Green SL (2010). The laboratory Xenopus sp.

[13] Ross AA, Rodrigues Hoffmann A, Neufeld JD (2019). The skin microbiome of vertebrates. Microbiome.

[14] Albecker MA, Belden LK, McCoy MW (2018). Comparative analysis of anuran amphibian skin microbiomes across inland and coastal wetlands. Microb Ecol.

[15] Duellman WE, William E, Trueb L (1994). Biology of amphibians.

[16] Abarca JG, Vargas G, Zuniga I, Whitfield SM, Woodhams DC, Kerby J, McKenzie VJ, Murillo-Cruz C, Pinto-Tomás AA (2018). Assessment of bacterial communities associated with the skin of Costa Rican amphibians at la Selva biological station. Front Microbiol.

[17] Lopes NP, Andrade LE, Prado BM, Haddad CFB, Pupo MT, Palacios-Rodríguez P, Brunetti AE, Melo WGP, Lyra ML (2019). Symbiotic skin bacteria as a source for sex-specific scents in frogs. Proc Natl Acad Sci.

[18] Kueneman JG, Parfrey LW, Woodhams DC, Archer HM, Knight R, McKenzie VJ (2014). The amphibian skin-associated microbiome across species, space and life history stages. Mol Ecol.

[19] Kueneman JG, Weiss S, McKenzie VJ (2017). Composition of micro-eukaryotes on the skin of the cascades frog (Rana cascadae) and patterns of correlation between skin microbes and Batrachochytrium dendrobatidis. Front Microbiol.

[20] Bell SC, Garland S, Alford RA (2018). Increased numbers of culturable inhibitory bacterial taxa may mitigate the effects of Batrachochytrium dendrobatidis in Australian wet tropics frogs. Front Microbiol.

[21] Ellison S, Knapp RA, Sparagon W, Swei A, Vredenburg VT (2018). Reduced skin bacterial diversity correlates with increased pathogen infection intensity in an endangered amphibian host. Mol Ecol.

[22] Weitzman CL, Gibb K, Christian K (2018). Skin bacterial diversity is higher on lizards than sympatric frogs in tropical Australia. PeerJ.

[23] Stecher B, Hardt W-D (2008). The role of microbiota in infectious disease. Trends Microbiol.

[24] Becker MH, Walke JB, Cikanek S, Savage AE, Mattheus N, Santiago CN, Minbiole KPC, Harris RN, Belden LK, Gratwicke B (2015). Composition of symbiotic bacteria predicts survival in Panamanian golden frogs infected with a lethal fungus. Proc R Soc B Biol Sci.

[25] Harrison XA, Price SJ, Hopkins K, Leung WTM, Sergeant C, Garner TWJ (2017). Host microbiome richness predicts resistance to disturbance by pathogenic infection in a vertebrate host. bioRxiv.

[26] Pearl EJ, Grainger RM, Guille M, Horb ME (2012). Development of xenopus resource centers: The National Xenopus Resource and the European Xenopus Resource Center. Genesis.

[27] Trott KA, Stacy BA, Lifland BD, Diggs HE, Harland RM, Khokha MK, Grammer TC, Parker JM (2004). Characterization of a mycobacterium ulcerans-like infection in a colony of African tropical clawed frogs (Xenopus tropicalis). Comp Med.

[28] Horb M, Wlizla M, Abu-Daya A, McNamara S, Gajdasik D, Igawa T, Suzuki A, Ogino H, Noble A, Nicolas M, Lafond T, Boujard D, Audic Y, Guillet B, Kashiwagi A, Kashiwagi K, Suzuki N, Tazawa I, Ochi H (2019). Xenopus resources: transgenic, inbred and mutant animals, training opportunities, and web-based support. Front Physiol.

[29] Wlizla M, McNamara S, Horb ME (2018). Generation and care of *Xenopus laevis* and Xenopus tropicalis embryos. Methods in molecular biology.

[30] The biology of xenopus - R. C. Tinsley, H. R. Kobel - Oxford University Press. https://global.oup.com/academic/product/the-biology-of-xenopus-9780198549741?cc=gb&lang=en&.

[31] Martel A, Boyen F, Bletz MC, Vences M, Bert W, Steinfartz S, Sabino-Pinto J, Bales E, Kelly M, Pasmans F, Van Praet S (2018). Disruption of skin microbiota contributes to salamander disease. Proc R Soc B Biol Sci.

[32] Bletz MC, Loudon AH, Becker MH, Bell SC, Woodhams DC, Minbiole KPC, Harris RN (2013). Mitigating amphibian chytridiomycosis with bioaugmentation: characteristics of effective probiotics and strategies for their selection and use. Ecol Lett.

[33] Piovia-Scott J, Rejmanek D, Woodhams DC, Worth SJ, Kenny H, McKenzie V, Lawler SP, Foley JE (2017). Greater species richness of bacterial skin symbionts better suppresses the amphibian fungal pathogen Batrachochytrium Dendrobatidis. Microb Ecol.

[34] Bitschar K, Sauer B, Focken J, Dehmer H, Moos S, Konnerth M, Schilling NA, Grond S, Kalbacher H, Kurschus FC, Götz F, Krismer B, Peschel A, Schittek B (2019). Lugdunin amplifies innate immune responses in the skin in synergy with host- and microbiota-derived factors. Nat Commun.

[35] Pace NR (1997). A molecular view of microbial diversity and the biosphere. Science.

[36] Amann RI, Ludwig W, Schleifer KH (1995). Phylogenetic identification and in situ detection of individual microbial cells without cultivation. Microbiol Rev.

[37] Knutie SA, Wilkinson CL, Kohl KD, Rohr JR (2017). Early-life disruption of amphibian microbiota decreases later-life resistance to parasites. Nat Commun.

[38] Estrada A, Hughey MC, Medina D, Rebollar EA, Walke JB, Harris RN, Belden LK (2019). Skin bacterial communities of neotropical treefrogs vary with local environmental conditions at the time of sampling. PeerJ.

[39] Colombo BM, Scalvenzi T, Benlamara S, Pollet N (2015). Microbiota and mucosal immunity in amphibians. Front Immunol.

[40] Antwis RE, Haworth RL, Engelmoer DJP, Ogilvy V, Fidgett AL, Preziosi RF (2014). Ex situ diet influences the bacterial community associated with the skin of red-eyed tree frogs (agalychnis callidryas). PLoS One.

[41] Walke JB, Becker MH, Loftus SC, House LL, Cormier G, Jensen RV, Belden LK (2014). Amphibian skin may select for rare environmental microbes. ISME J.

[42] Rebollar EA, Hughey MC, Medina D, Harris RN, Ibáñez R, Belden LK (2016). Skin bacterial diversity of Panamanian frogs is associated with host susceptibility and presence of Batrachochytrium dendrobatidis. ISME J.

[43] Passos LF, Garcia G, Young RJ (2018). Comparing the bacterial communities of wild and captive golden mantella frogs: implications for amphibian conservation. PLoS One.

[44] Woodhams DC, Kueneman JG, McKenzie VJ, Archer HM, Harris R, Knight R (2016). Probiotic treatment restores protection against lethal fungal infection lost during amphibian captivity. Proc R Soc B Biol Sci.

[45] Altschul SF, Gish W, Miller W, Myers EW, Lipman DJ (1990). Basic local alignment search tool. J Mol Biol.

[46] Ross DV, Suzina NE, Gafarov AB, Machulin AV, Esikova TZ, Shorokhova AP, Duda VI, Boronin AM (2019). Characterization of ultrasmall chryseobacterium strains FM1 and FM2 isolated from *Xenopus laevis* skin. Microbiology.

[47] Kostanjšek R, Prodan Y, Stres B, Trontelj P (2019). Composition of the cutaneous bacterial community of a cave amphibian, *Proteus anguinus*. FEMS Microbiol Ecol.

[48] Bates KA, Clare FC, O’Hanlon S, Bosch J, Brookes L, Hopkins K, McLaughlin EJ, Daniel O, Garner TW, Fisher MC, Harrison XA (2018). Amphibian chytridiomycosis outbreak dynamics are linked with host skin bacterial community structure. Nat Commun.

[49] Bletz MC, Perl RGB, Bobowski BTC, Japke LM, Tebbe CC, Dohrmann AB, Bhuju S, Geffers R, Jarek M, Vences M (2017). Amphibian skin microbiota exhibits temporal variation in community structure but stability of predicted Bd-inhibitory function. ISME J.

[50] Longo AV, Zamudio KR (2017). Environmental fluctuations and host skin bacteria shift survival advantage between frogs and their fungal pathogen. ISME J.

[51] Berry DL, Schwartzman R and Brown D D. Metamorphosis: The Eighth Symposium of the British Society for Developmental... - British Society for Developmental Biology. Symposium, Senior Lecturer Department of Human Morphology Michael Balls, British Society for Developmental Biology - Google Books. 59–87 https://books.google.co.uk/books/about/Metamorphosis.html?id=8hAyAAAAMAAJ&redir_esc=y (1985).

[52] Hernández-Gómez O, Hoverman JT, Williams RN (2017). Cutaneous microbial community variation across populations of eastern hellbenders (Cryptobranchus alleganiensis alleganiensis). Front Microbiol.

[53] Bird AK, Prado-Irwin SR, Vredenburg VT, Zink AG (2018). Skin microbiomes of California terrestrial salamanders are influenced by habitat more than host phylogeny. Front Microbiol.

[54] Xie ZY, Zhou YC, Wang SF, Mei B, Xu XD, Wen WY, Feng YQ (2009). First isolation and identification of Elizabethkingia meningoseptica from cultured tiger frog, Rana tigerina rugulosa. Vet Microbiol.

[55] Ilardi P, Fernández J, Avendaño-Herrera R (2009). Chryseobacterium piscicola sp. nov., isolated from diseased salmonid fish. Int J Syst Evol Microbiol.

[56] Kirk KE, Hoffman JA, Smith KA, Strahan BL, Failor KC, Krebs JE, Gale AN, Do TD, Sontag TC, Batties AM, Mistiszyn K, Newman JD (2013). Chryseobacterium angstadtii sp. nov., isolated from a newt tank. Int J Syst Evol Microbiol.

[57] Rebollar EA, Gutiérrez-Preciado A, Noecker C, Eng A, Hughey MC, Medina D, Walke JB, Borenstein E, Jensen RV, Belden LK, Harris RN (2018). The skin microbiome of the neotropical frog Craugastor fitzingeri: inferring potential bacterial-host-pathogen interactions from metagenomic data. Front Microbiol.

[58] Jiménez RR, Alvarado G, Estrella J, Sommer S (2019). Moving beyond the host: unraveling the skin microbiome of endangered Costa Rican amphibians. Front Microbiol.

[59] Tinsley RC, Coxhead PG, Stott LC, Tinsley MC, Piccinni MZ, Guille MJ (2015). Chytrid fungus infections in laboratory and introduced *Xenopus laevis* populations: assessing the risks for U.K. native amphibians. Biol Conserv.

[60] Robertson SJ, Lemire P, Maughan H, Goethel A, Turpin W, Bedrani L, Guttman DS, Croitoru K, Girardin SE, Philpott DJ (2019). Comparison of Co-housing and littermate methods for microbiota standardization in mouse models. Cell Rep.

[61] Ma BW, Bokulich NA, Castillo PA, Kananurak A, Underwood MA, Mills DA, Bevins CL (2012). Routine habitat change: a source of unrecognized transient alteration of intestinal microbiota in laboratory mice. PLoS One.

[62] Katsnelson A (2019). Minding the microbiome of your mice. Lab Anim.

[63] Leystra AA, Clapper ML (2019). Gut microbiota influences experimental outcomes in mouse models of colorectal cancer. Genes.

[64] Klindworth A, Pruesse E, Schweer T, Peplies J, Quast C, Horn M, Glöckner FO (2013). Evaluation of general 16S ribosomal RNA gene PCR primers for classical and next-generation sequencing-based diversity studies. Nucleic Acids Res.

[65] Babraham bioinformatics - FastQC a quality control tool for high throughput sequence data. http://www.bioinformatics.babraham.ac.uk/projects/fastqc/.

[66] Babraham bioinformatics - trim galore! https://www.bioinformatics.babraham.ac.uk/projects/trim_galore/.

[67] Caporaso JG, Kuczynski J, Stombaugh J, Bittinger K, Bushman FD, Costello EK, Fierer N, Pẽa AG, Goodrich JK, Gordon JI, Huttley GA, Kelley ST, Knights D, Koenig JE, Ley RE, Lozupone CA, McDonald D, Muegge BD, Pirrung M (2010). QIIME allows analysis of high-throughput community sequencing data. Nat Methods.

[68] Quast C, Pruesse E, Yilmaz P, Gerken J, Schweer T, Yarza P, Peplies J, Glöckner FO (2013). The SILVA ribosomal RNA gene database project: improved data processing and web-based tools. Nucleic Acids Res.

[69] McMurdie PJ, Holmes S (2013). Phyloseq: an R package for reproducible interactive analysis and graphics of microbiome census data. PLoS One.

[70] Magurran AE (1988). Ecological diversity and its measurement.

[71] Bray JR, Curtis JT (1957). An ordination of the upland forest communities of Southern Wisconsin. Ecol Monogr.

[72] Love MI, Huber W, Anders S (2014). Moderated estimation of fold change and dispersion for RNA-seq data with DESeq2. Genome Biol.

[73] Benjamini Y, Hochberg Y (1995). Controlling the false discovery rate: a practical and powerful approach to multiple testing. J R Stat Soc Ser B Methodol.

[74] Wickham H (2016). ggplot2. Springer.

[75] CRAN - package pheatmap. http://cran.nexr.com/web/packages/pheatmap/index.html.

[76] Oksanen J, Blanchet FG, Friendly M, Kindt R, Legendre P, Mcglinn D, Minchin PR, O’hara RB, Simpson GL, Solymos P, Henry M, Stevens H, Szoecs E, Maintainer HW (2019). Package ‘vegan’ title community ecology package version 2.5–6.

