# Supplementary material for: The skin microbiome of Xenopus laevis and the effects of husbandry conditions
Source: Anim Microbiome. 2021 Feb 5;3:17. doi: 10.1186/s42523-021-00080-w (PMC7866774; doi:10.1186/s42523-021-00080-w)
Supplement: Supplementary file 1 — Additional file 1: Supplementary Figures: Additional figures. [file 42523_2021_80_MOESM1_ESM.pdf]

Supplementary Figures

Supplementary Figure 1: Sample collection and processing workflow. Workflow detailing the sample collection, processing, DNA extraction and sequencing steps performed during this experiment.

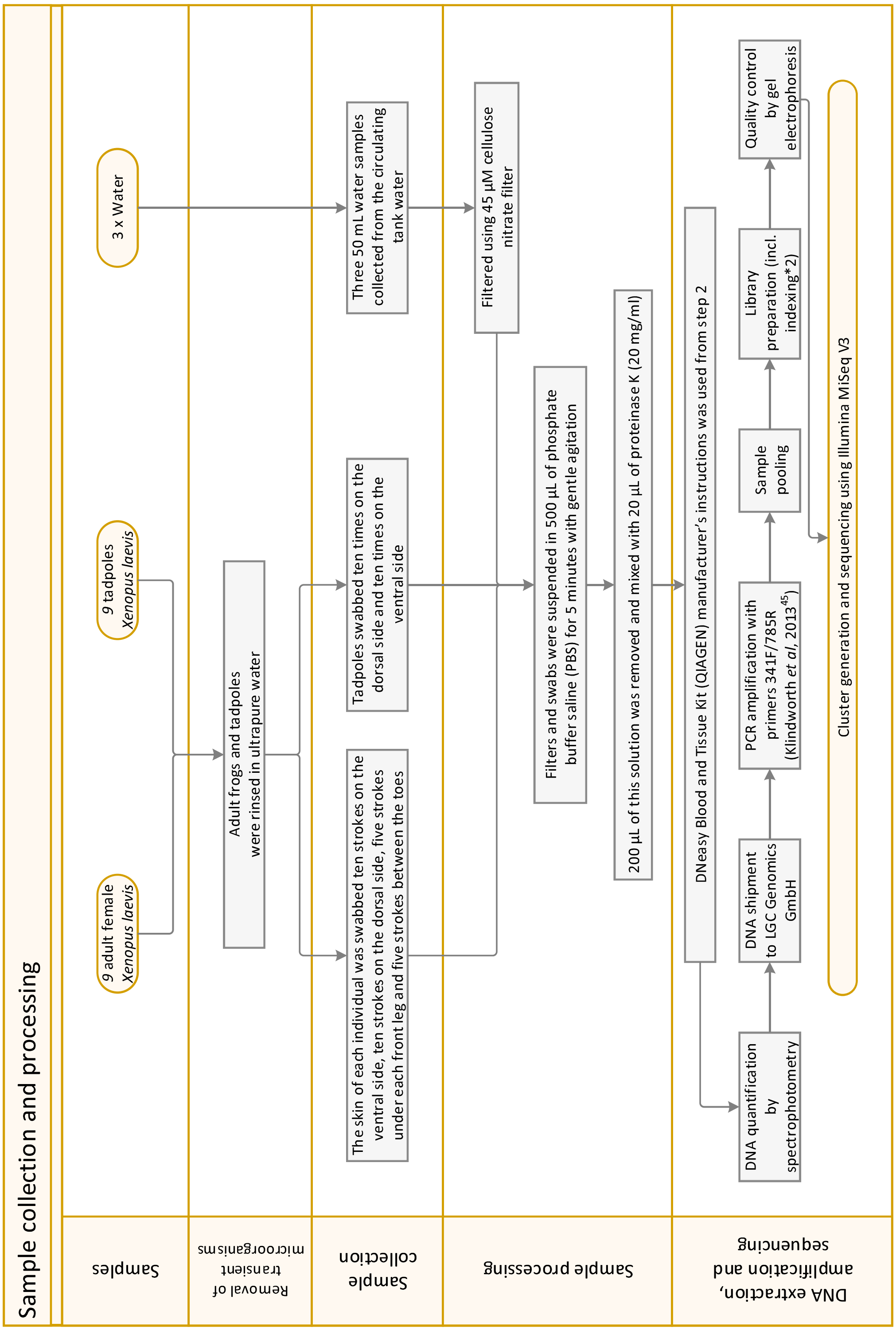

Supplementary Figure 2: Rarefaction curve showing the change in diversity assessed from subsets of data. Curves showing the diversity (assessed based on the Shannon Index) when taking random subsets of the data. Asymptotic rarefaction curves suggest saturation of diversity estimates from the data available, and indicate that coverage is suitable to capture the full abundance of bacterial populations. Read depth for subsampling was limited to a maximum of 20,000 OTUs.

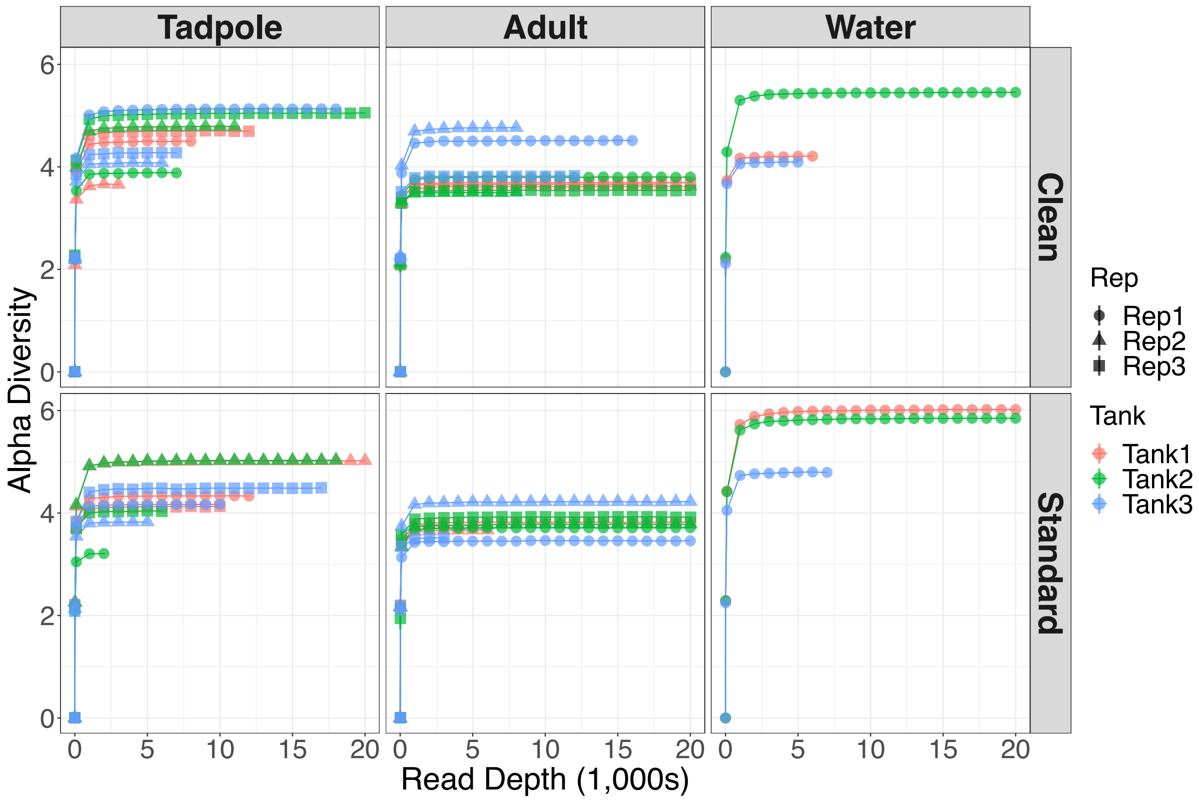

Supplementary Figure 3: Barplot showing the distribution of bacteria at the genus level of taxonomy. In each case, the top 15 genera are highlighted, with all remaining genera shown in grey.

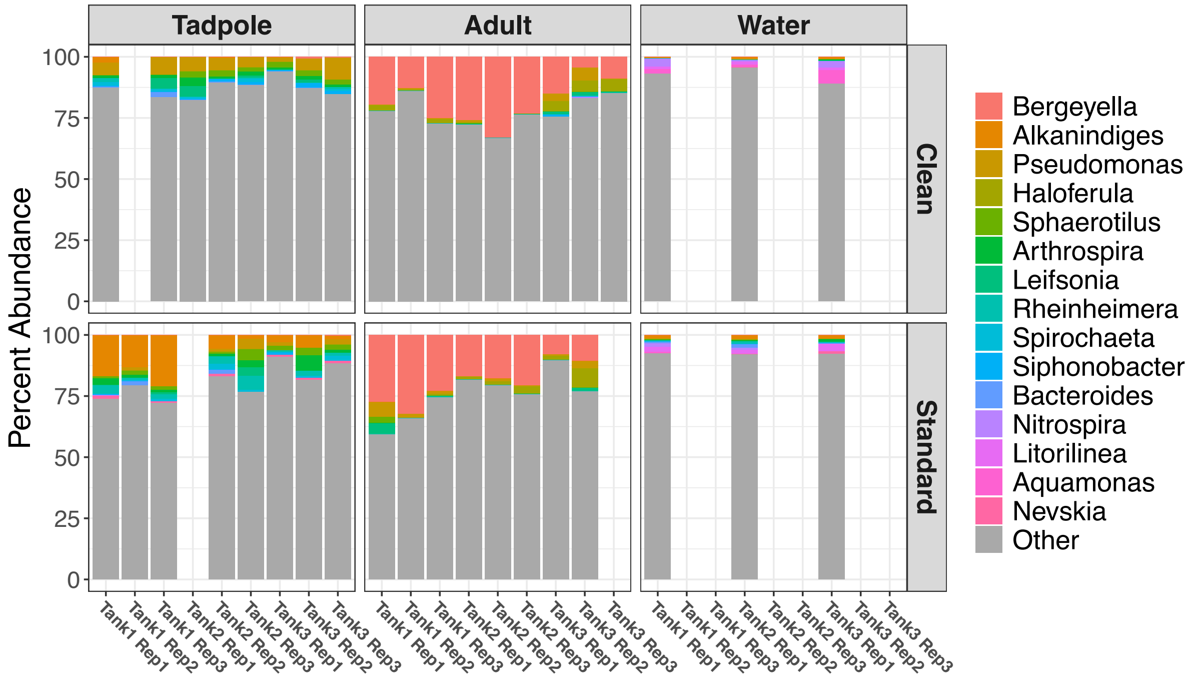

Supplementary Figure 4: Phylogeny of Core Microbiome. Phylogenetic tree for the OTUs identified as falling under one of the 20 genera identified as being greater than 1% abundant in both the clean and standard conditions for either adult *Xenopus laevis*, tadpoles, or environmental water samples. Samples are coloured based on their group (clean, red; standard, blue) with shapes representing the sample in which the OTU was identified (tadpole, circle; adult frog, triangle; water, square). The size of each point is scaled relative to its percentage abundance (on a log 2 scale). Groupings for each of the 20 genera are annotated.

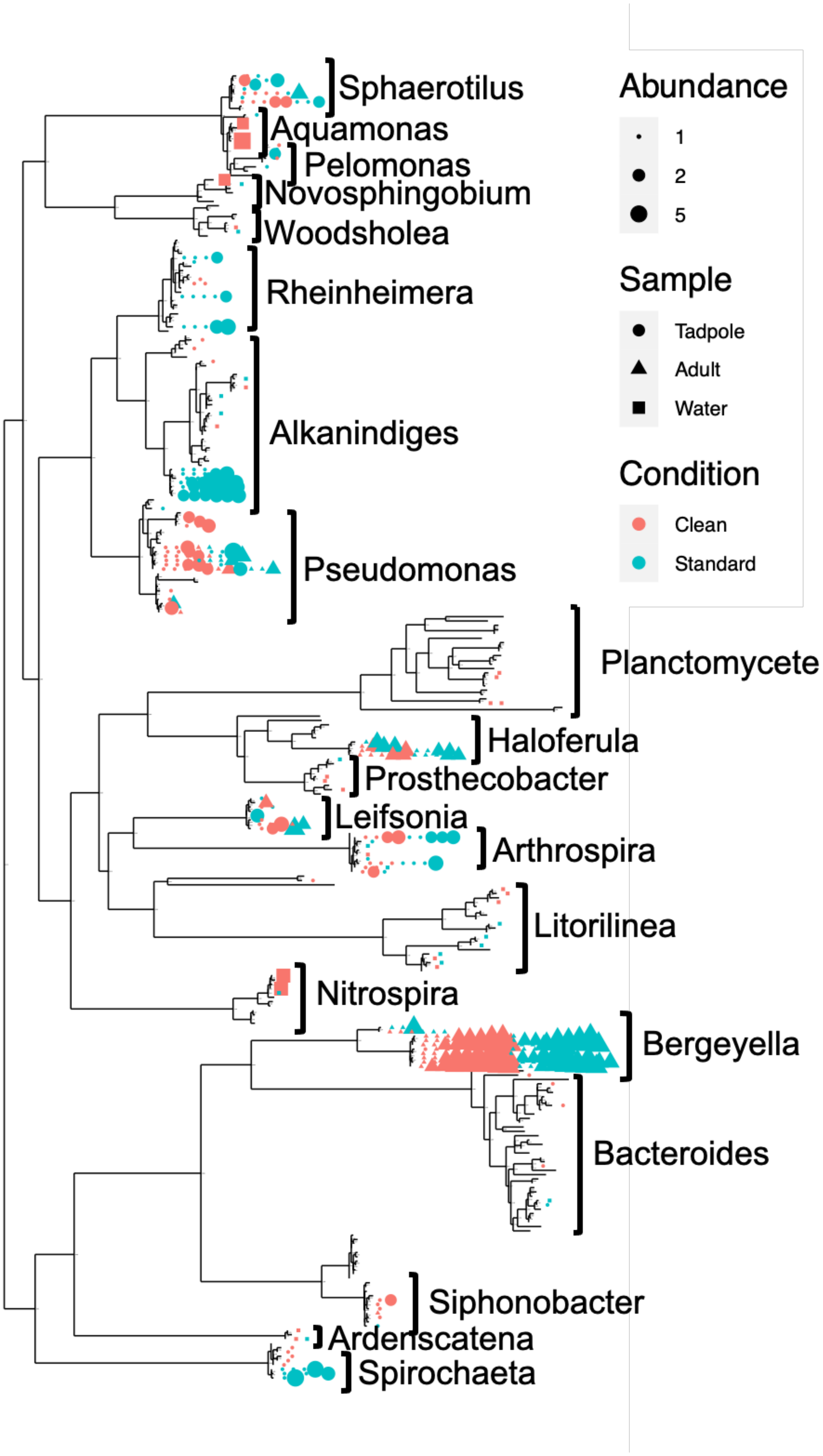

Sphaerotilus
Aquamonas
Pelomonas
Novosphingobium
Woodsholea
Rheinheimera
Alkanindiges
Pseudomonas
Planctomycete
Haloferula
Prosthecobacter
Leifsonia
Arthrospira
Litorilinea
Nitrospira
Bergeyella
Bacteroides
Siphonobacter
Ardenscatena
Spirochaeta
Abundance
1
2
5
Sample
Tadpole
Adult
Water
Condition
Clean
Standard
